# Cardiorenal Safety Markers With Injectable Glucagon-Like Peptide-1 (GLP-1) Agonists in Type 2 Diabetes: A Network Meta-Analysis

**DOI:** 10.7759/cureus.96162

**Published:** 2025-11-05

**Authors:** Abigail Rai, Mahesh Kolli, Gaurav Pratap Singh, Rishu, Chao Yuan Li Cai, Hariharasudhan Balaji, Bendangjungla Pongen, Sandra Joy, Shahid Sai A.K.

**Affiliations:** 1 Acute Medical Unit (AMU), Impur Christian Hospital, Mokokchung, IND; 2 Telemedicine, Apollo Hospitals, Chennai, IND; 3 Critical Care Medicine, King's College Hospital NHS Foundation Trust, London, GBR; 4 General Internal Medicine, Dudley Group of NHS Foundation Trust, Dudley, GBR; 5 General Medicine, Hywel Dda Health Board, Wales, GBR; 6 Rotational in Medicine, Hywel Dda Health Board, Wales, GBR; 7 Emergency Medicine, Southern Health and Social Care Trust, Armagh, GBR; 8 Emergency Medicine, Bethesda Hospital, Kohima, IND; 9 Neurology, Baby Memorial Hospital, Kozhikode, IND; 10 Emergency Medicine, Baby Memorial Hospital, Thodupuzha, IND

**Keywords:** cardio protection, glucagon-like peptide-1 receptor agonist, network-meta analysis, renal safety, types 2 diabetes

## Abstract

Type 2 diabetes mellitus (T2DM) markedly increases the risk of cardiovascular and renal complications, emphasizing the need for therapies that extend benefits beyond glycemic control. Injectable glucagon-like peptide-1 receptor agonists (GLP-1 RAs) have demonstrated both cardioprotective and renoprotective potential, but the comparative efficacy of individual agents remains uncertain. This network meta-analysis (NMA) evaluated and ranked the effects of GLP-1 RAs on major adverse cardiovascular events (MACE) and renal composite outcomes, both prespecified as co-primary endpoints. A systematic search of PubMed, Scopus, ScienceDirect, Web of Science, and the Cochrane Library was conducted from inception to January 2024. Eligible randomized controlled trials enrolled adults with T2DM, including those with or without established cardiovascular disease or chronic kidney disease. Renal composite outcomes were defined as a decline in estimated glomerular filtration rate (eGFR) and/or onset of macroalbuminuria. Direct and indirect evidence were synthesized using a frequentist NMA framework to generate odds ratios (ORs) with 95% confidence intervals (CIs), while treatment hierarchy was assessed with Surface Under the Cumulative Ranking Curve (SUCRA) values. Transitivity and consistency assumptions were tested and satisfied, and the risk of bias was assessed with the Cochrane RoB2 tool. Fifteen randomized trials involving a pooled sample of more than 90,000 participants were included. Efpeglenatide ranked highest for both MACE (OR: 0.74; 95% CI: 0.62-0.87; SUCRA: 81.5%) and renal outcomes (OR: 0.68; 95% CI: 0.57-0.81; SUCRA: 81.33%), underscoring its clinical significance over other effective agents such as albiglutide and semaglutide. Albiglutide, semaglutide, dulaglutide, and liraglutide also provided significant benefits, albeit with lower rankings. No major inconsistency or publication bias was detected. In conclusion, this NMA reinforces the class-wide cardiorenal benefits of GLP-1 receptor agonists while emphasizing variations in efficacy among individual agents, the limited evidence base for certain drugs, and the need for future head-to-head trials specifically designed to evaluate cardiovascular and renal endpoints.

## Introduction and background

Type 2 diabetes mellitus (T2DM) represents a significant global health burden, with patients facing substantially elevated risks for cardiovascular disease, including myocardial infarction, stroke, heart failure, and cardiovascular death [1]. The incretin-based therapies have revolutionized diabetes care by targeting multiple pathophysiological pathways, including glucose-dependent insulin secretion, glucagon suppression, and gastric motility regulation [2]. Over the past decade, extensive cardiovascular outcomes trials (CVOTs) have established the cardioprotective effects of several GLP-1 RAs, with meta-analyses consistently demonstrating a 12%-14% reduction in major adverse cardiovascular events (MACE) compared to placebo [3,4].

The cardiovascular benefits of GLP-1 RAs appear to extend beyond their antihyperglycemic effects, with emerging evidence suggesting direct cardioprotective mechanisms including anti-inflammatory, anti-atherosclerotic, and pleiotropic vascular effects [5]. However, significant heterogeneity exists within the GLP-1 RA class, with individual agents demonstrating varying degrees of cardiovascular efficacy [6]. This variability suggests distinct pharmacological properties and clinical profiles within the class, necessitating comparative effectiveness research to inform optimal therapeutic selection [7]. Concurrent with cardiovascular benefits, GLP-1 RAs have demonstrated promising renal protective effects, with recent meta-analyses revealing significant reductions in clinically important kidney events and kidney failure [8,9]. The renoprotective mechanisms appear multifaceted, involving direct effects on renal hemodynamics, anti-inflammatory pathways, and antifibrotic processes [10]. These findings are particularly relevant given the substantial burden of diabetic kidney disease and the urgent need for interventions that can slow progression to end-stage renal disease [11]. Inter-agent heterogeneity between GLP-1 RAs likely reflects differences in trial populations, baseline cardiovascular and renal risk, variations in outcome definitions, and potential molecular distinctions among GLP-1 RAs [6]. This variability underscores the need for comparative assessment across agents.

While meta-analyses have consistently confirmed the overall benefit of GLP-1 RAs in reducing MACE with a 12%-14% relative reduction [12,13], the relative ranking and differential impact of each agent on both cardiovascular and renal outcomes remain unclear. Importantly, renal outcomes are inconsistently reported across trials and often as secondary endpoints, limiting the precision and clinical translation of findings in real-world nephroprotective decision-making. Renal composite outcomes in previous GLP-1 RA trials have typically included progression of albuminuria, sustained decline in estimated glomerular filtration rate (eGFR), and, in some cases, kidney failure [14,15]. This inconsistency limits the clinical translation of trial findings to nephroprotective strategies in routine care. Direct head-to-head comparisons of GLP-1 RAs are lacking, precluding reliable relative efficacy assessments from individual trials. Network meta-analysis (NMA) allows integration of both direct and indirect evidence to generate relative treatment rankings, thereby addressing this critical gap. This study addresses these key gaps by employing a robust NMA framework to synthesize both direct and indirect evidence, enabling comparative effectiveness and safety profiling of all major injectable GLP-1 RAs, namely, liraglutide, semaglutide, dulaglutide, albiglutide, efpeglenatide, exenatide (once weekly), and lixisenatide. The primary objectives are to evaluate the relative efficacy of GLP-1 RAs in reducing MACE, to assess and rank agents according to their effects on renal composite outcomes, and to identify agents with the most favorable cardiovascular-renal risk profile.

## Review

Methodology

This study was conducted in accordance with the Preferred Reporting Items for Systematic Reviews and Meta-Analyses (PRISMA) for network meta-analysis (NMA) guidelines [16] for conducting systematic reviews and meta-analyses. Compliance with the principles of the Declaration of Helsinki further reinforced the ethical foundation of this review. This review exclusively analyzed secondary data from existing studies and, therefore, qualifies for exemption from informed consent or institutional review board approval. The study protocol was registered on the PROSPERO (International Prospective Register of Systematic Reviews) online database with the registration number CRD420251063458.

Search Strategy

The search strategy was developed by two authors (AR and MK) according to the specified criteria. A comprehensive literature search was conducted across five major electronic databases: PubMed, Cochrane Library, ScienceDirect, Web of Science, and Scopus, to identify studies evaluating the cardiovascular (CV) and renal outcomes of glucagon-like peptide-1 receptor agonists (GLP-1 RAs) in patients with T2DM till January 2025. The final database search was conducted on January 15, 2025. Boolean operators and controlled vocabulary (e.g., MeSH terms) were used to combine keywords related to GLP-1 RAs (e.g., “liraglutide,” “semaglutide,” “exenatide”), type 2 diabetes (“T2DM,” “type 2 diabetes mellitus”), and relevant clinical outcomes (e.g., “major adverse cardiovascular events,” “MACE,” “renal outcomes,” “nephropathy”). The search strategy was tailored to the syntax and indexing of each database to maximize sensitivity. Filters were applied to include only human studies published in English. No restriction was applied to the publication year to ensure broad coverage. A detailed search strategy is given in Table 1.

**Table 1:** Search strategy. GLP-1 RA, glucagon-like peptide-1 receptor agonist; T2DM, type 2 diabetes mellitus; MACE, major adverse cardiovascular events; MeSH, Medical Subject Headings; TS, topic search

Data	Key Words
PubMed	("Glucagon-Like Peptide 1 Receptor Agonists"[MeSH] OR "GLP-1 receptor agonist" OR "Semaglutide" OR "Liraglutide" OR "Dulaglutide" OR "Exenatide" OR "Albiglutide" OR "Efpeglenatide" OR "Cotadutide" OR "Lixisenatide") AND ("Diabetes Mellitus, Type 2"[MeSH] OR "Type 2 Diabetes" OR "T2DM") AND ("Cardiovascular Diseases"[MeSH] OR "Major Adverse Cardiovascular Events" OR "MACE" OR "myocardial infarction" OR "stroke" OR "cardiovascular death")
Scopus	TITLE-ABS-KEY("GLP-1 receptor agonist" OR "semaglutide" OR "liraglutide" OR "dulaglutide" OR "exenatide" OR "albiglutide" OR "efpeglenatide" OR "cotadutide" OR "lixisenatide") AND TITLE-ABS-KEY("type 2 diabetes" OR "T2DM") AND TITLE-ABS-KEY("MACE" OR "major adverse cardiovascular events" OR "cardiovascular outcomes" OR "myocardial infarction" OR "stroke" OR "cardiovascular death")
Cochrane Library	("Glucagon-Like Peptide 1 Receptor Agonists" OR "GLP-1 receptor agonist" OR "Semaglutide" OR "Liraglutide" OR "Dulaglutide" OR "Exenatide" OR "Albiglutide" OR "Efpeglenatide" OR "Cotadutide" OR "Lixisenatide") AND ("Diabetes Mellitus, Type 2" OR "Type 2 Diabetes" OR "T2DM") AND ("Cardiovascular Diseases" OR "Major Adverse Cardiovascular Events" OR "MACE" OR "myocardial infarction" OR "stroke" OR "cardiovascular death")
ScienceDirect	(glucagon-like peptide-1 receptor agonist OR GLP-1 agonist) AND (type 2 diabetes OR T2DM) AND ((MACE) OR [major adverse cardiovascular events] OR (cardiovascular outcomes) OR (myocardial infarction) OR (Renal failure))
Web of Science	TS=("GLP-1 receptor agonist" OR "glucagon-like peptide-1 receptor agonist" OR "GLP-1RA" OR "liraglutide" OR "semaglutide" OR "dulaglutide" OR "exenatide" OR "albiglutide" OR "lixisenatide" OR "efpeglenatide") AND TS=("type 2 diabetes" OR "T2DM") AND TS=("major adverse cardiovascular events" OR "MACE" OR "cardiovascular outcomes" OR "myocardial infarction" OR "stroke" OR "cardiovascular death") AND TS=("renal outcomes" OR "kidney disease" OR "nephropathy" OR "renal adverse events")

Study Selection

A comprehensive and structured approach was employed to select studies evaluating the CV and renal outcomes associated with GLP-1 RAs in patients with T2DM. The selection process followed the PICO framework to ensure consistency and relevance across the identified literature.

Population (P): Studies included adult participants (≥18 years) with a confirmed diagnosis of T2DM, with or without established cardiovascular disease (CVD), heart failure (HF), or chronic kidney disease (CKD). Studies involving both primary and secondary prevention cohorts were eligible. Populations with acute coronary syndromes, recent hospitalization, or undergoing cardiac surgery were also included if T2DM was a common characteristic.

Intervention (I): The intervention of interest was any approved or investigational injectable GLP-1 RA, including dulaglutide, liraglutide, semaglutide, albiglutide, efpeglenatide, exenatide, or lixisenatide, administered as monotherapy.

Comparator (C): Eligible comparators included placebo, standard care, or other active glucose-lowering therapies (e.g., sitagliptin or insulin). Trials were eligible whether GLP-1 RAs were administered as monotherapy or as add-on therapy, provided they reported CV or renal outcomes with a defined comparator arm. Early-phase or single-arm pharmacokinetic/pharmacodynamic studies without these outcomes were excluded.

Outcomes (O): Included studies must report on at least one of the following: major adverse cardiovascular events (MACE), CV mortality, HF events, renal composite outcomes (e.g., decline in eGFR, onset of macroalbuminuria), or adverse events. Secondary outcomes such as HbA1c reduction, body weight, or quality of life were noted when relevant. Studies were excluded if they involved type 1 diabetes mellitus, animal models, in vitro experiments, or lacked a clear comparator. Reviews, editorials, case reports, and conference abstracts without full data were also excluded. Studies without a comparator group were excluded unless pharmacokinetic or pharmacodynamic endpoints were central to early-phase trials.

Data Collection and Quality Evaluation

Data collection and quality evaluation were carried out independently by two reviewers, who screened the titles and abstracts of studies in EndNote 20.2.1 based on the inclusion criteria. Full texts of potentially eligible studies were reviewed, with disagreements resolved through decisioning by a blinded third author. Data were extracted into a pre-tested Microsoft Excel sheet for consistency, documenting key details such as author, year, study design, diagnosis, sample size, interventions and measured outcomes. Renal outcomes were harmonized to address variations in endpoint definitions across studies. All renal adverse events included in this analysis were directly reported in the original RCTs, not derived or reconstructed from secondary parameters. When multiple renal endpoints were presented, the most comprehensive composite renal measure was selected typically including new or worsening macroalbuminuria, sustained eGFR decline (≥30% to 40%), doubling of serum creatinine, or initiation of renal replacement therapy. These events were standardized as binary outcomes (event vs. no event) based on trial-specific definitions, consistent with previous meta-analyses on GLP-1 RA renal outcomes. Methodological rigor and potential biases were also documented to ensure systematic data collection and analysis. For RCTs, the Cochrane ROB2 tool was used [17], to assess the ROB associated with the included studies for quality appraisal. Each study was evaluated across five domains (D1-D5) as per Cochrane’s RoB 2.0 tool. Results were visualized using the RoB Visualization tool, with traffic light plots and summary bar charts [18]. To minimize biases, rigorous inclusion criteria, consistent data extraction, and sensitivity analyses were applied. Data on concomitant cardioprotective therapies, including baseline use of SGLT2 inhibitors and insulin, were extracted qualitatively from published trial reports where available. However, due to lack of uniform reporting and absence of individual patient-level data, no formal subgroup analysis or meta-regression adjustment for these background therapies was conducted. The influence of background therapy was, therefore, considered narratively during result interpretation.

Statistical Analysis

Statistical analysis for this study was conducted using the MetaInsight web-based platform, a validated tool for performing NMA [19]. Both frequentist and Bayesian methods were utilized to ensure robustness and consistency of results. Initially, traditional pairwise meta-analyses were conducted to compute odds ratios (ORs) and 95% confidence intervals (CIs) for direct comparisons between interventions. Subsequently, a frequentist NMA was performed using a multivariate meta-analysis model that integrates both direct and indirect evidence across the treatment network, assuming a common heterogeneity variance. This approach enabled estimation of pooled ORs with 95% CIs for all possible treatment comparisons. To enhance interpretation and guide clinical decision-making, Bayesian NMA was conducted using Markov Chain Monte Carlo (MCMC) simulation, producing posterior distributions of treatment effects. Treatment hierarchy was assessed through SUCRA values, which quantify the probability of each treatment being the most effective or safest. SUCRA values summarize the probability of each treatment being ranked highest; they are point estimates without CIs. The corresponding rankograms already presented illustrate the degree of uncertainty around these rankings. SUCRA rankings should therefore be interpreted alongside the pooled ORs and 95% CIs, as overlapping CIs among top agents indicate that their relative ranks may not differ significantly. Model convergence and consistency were assessed via trace plots, Gelman-Rubin diagnostics, and node-splitting methods. Random-effects frequentist and Bayesian NMA models were applied to address heterogeneity. Renal endpoints were harmonized to the broadest composite event and standardized as binary outcomes. Inconsistency was evaluated using I² and node-splitting. Publication bias was examined using Egger’s and Begg’s tests alongside visual funnel plots, revealing potential bias in the residual biomaterial outcome. Sensitivity analyses were conducted by excluding studies with high risk or unclear bias. This comprehensive statistical framework ensured the robustness of comparative efficacy estimates and enhanced the reliability of the synthesized evidence across diverse biomaterial interventions.

Results

**Figure 1:**
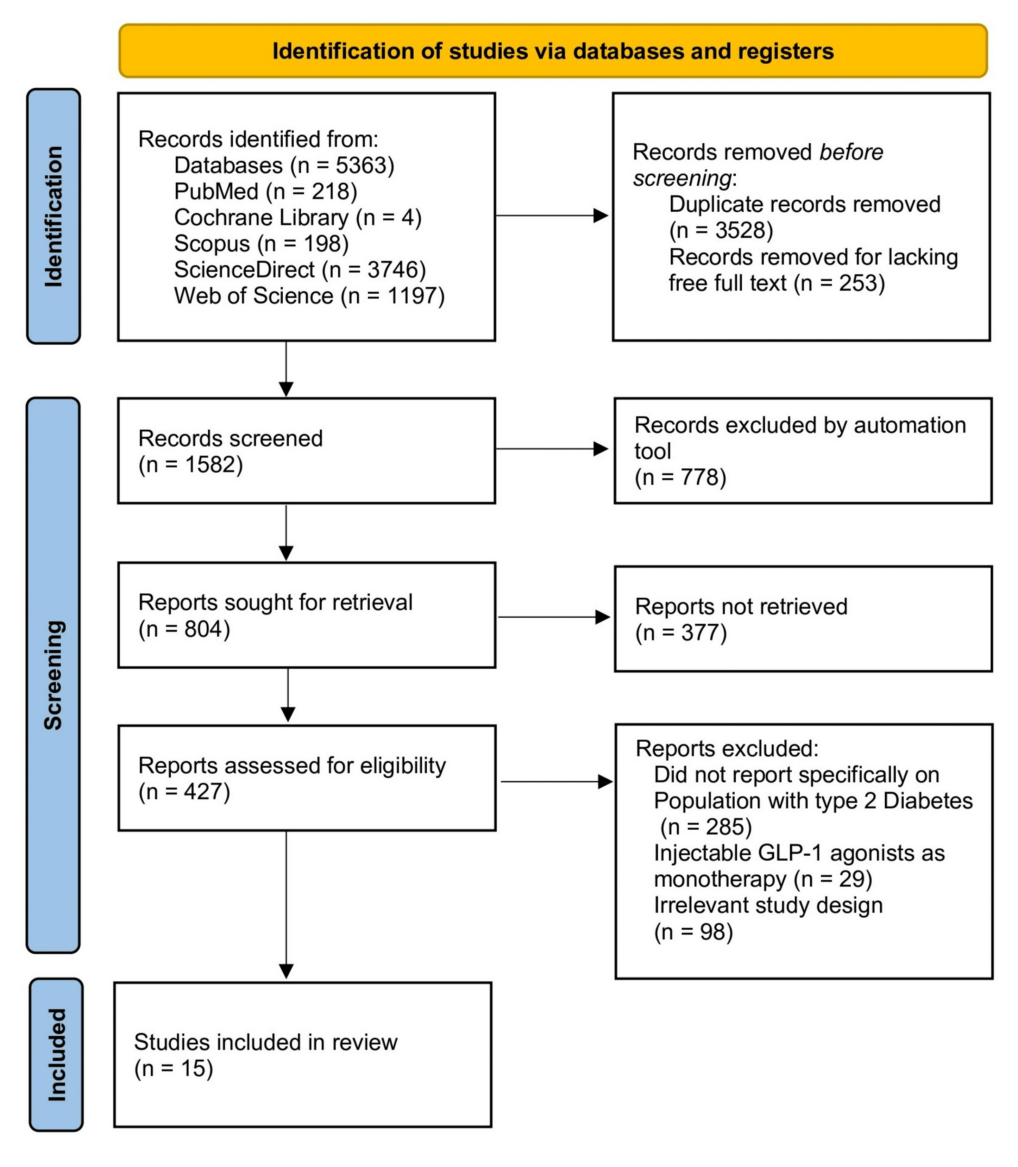
PRISMA flowchart. PRISMA, Preferred Reporting Items for Systematic Reviews and Meta-Analyses

The PRISMA flowchart in Figure 1 outlines the systematic process used to identify and select studies included in this review. Initially, a total of 5,363 records were identified across five electronic databases: PubMed (218), Cochrane Library (4), Scopus (198), ScienceDirect (3,746), and Web of Science (1,197). Before screening, 3,528 duplicate records were removed, along with 253 records that lacked free full-text availability, resulting in 1,582 records eligible for screening. During the screening phase, 778 records were excluded by an automation tool, leaving 804 reports for retrieval. Of 804 full-text reports sought, 377 could not be retrieved because they were conference abstracts without full text, contained broken links, or were behind paywalls. Mitigation efforts included registry searches (ClinicalTrials.gov, ISRCTN, EudraCT), interlibrary loans, author contact (12 attempts), and reference-list screening. The remaining 427 reports underwent full-text eligibility assessment. Among these, 285 were excluded for not reporting specifically on populations with T2DM, 29 were excluded for focusing on injectable GLP-1 agonists used as monotherapy rather than in combination or comparison, and 127 were excluded due to irrelevant study design (e.g., observational studies, reviews, or animal studies). Ultimately, 15 studies met the inclusion criteria and were included in the final systematic review.

Study Characteristics

The included studies span multinational and regional settings, as shown in Table 2, assessing GLP-1 RAs in T2DM populations, often with CV or renal comorbidities. Large trials such as REWIND, AMPLITUDE-O, LEADER, and SUSTAIN-6 evaluated the impact of agents like dulaglutide, efpeglenatide, liraglutide, and semaglutide on MACE, renal outcomes, and mortality. Dulaglutide and efpeglenatide consistently reduced MACE and renal deterioration across diverse populations, including patients on background SGLT2 inhibitors. Semaglutide showed substantial benefits in HF with preserved ejection fraction (HFpEF) but was linked with increased retinopathy risk in SUSTAIN-6. Trials like EXSCEL and ELIXA demonstrated CV safety but no superiority.

**Table 2:** Characteristics of the included studies. GLP-1 RA, glucagon-like peptide-1 receptor agonist; T2DM, type 2 diabetes mellitus; CVD, cardiovascular disease; CV, cardiovascular; MACE, major adverse cardiovascular events; MI, myocardial infarction; HR, hazard ratio; PY, person-years; HbA1c, glycated hemoglobin; FPG, fasting plasma glucose; AEs, adverse events; GI, gastrointestinal; eGFR, estimated glomerular filtration rate; RRT, renal replacement therapy; CKD, chronic kidney disease; HF, heart failure; HFpEF, heart failure with preserved ejection fraction; HFrEF, heart failure with reduced ejection fraction; RCT, randomized controlled trial; ASCVD, atherosclerotic cardiovascular disease; NT-proBNP, N-terminal pro–B-type natriuretic peptide; 6MWD, 6-minute walk distance; QoL, quality of life; BP, blood pressure; LDL, low-density lipoprotein; UACR, urine albumin-to-creatinine ratio; ACS, acute coronary syndrome; CI, confidence interval

Study	Country	Sample size	Diagnosis/population	Outcomes measured	Key findings
Dagenais et al., 2020 [20]	Multinational (part of REWIND)	9,901	Type 2 diabetes with CVD or CV risk factors	- Total MACE or non-CV deaths - Expanded MACE or non-CV deaths	Dulaglutide reduced total MACE or non-CV deaths (35.8 vs. 40.3 per 1000 PY; HR ~0.89–0.90, *P *< 0.05). Also reduced expanded MACE (67.1 vs. 74.7 per 1000 PY; HR ~0.90–0.93, *P *< 0.05).
Gadde et al., 2017 [21]	Multicenter (open-label)	365	Type 2 diabetes with suboptimal control on metformin	- HbA1c reduction - FPG - Body weight - AEs	Exenatide QWS-AI reduced HbA1c more than sitagliptin (−1.13% vs. −0.75%; *P* = 0.02) and placebo (*P* = 0.001). Greater % achieved HbA1c <7%. GI side effects and injection site reactions were common with exenatide.
Gerstein et al., 2019 (Renal analysis of REWIND) [22]	Multinational	9,901	Type 2 diabetes with CVD or CV risk factors	- Composite renal outcome (macroalbuminuria, eGFR decline ≥30%, RRT)	Dulaglutide reduced renal outcomes (HR 0.85; *P* = 0.0004), especially new macroalbuminuria (HR 0.77; *P* < 0.0001). eGFR decline and RRT did not reach statistical significance.
Gerstein et al., 2019 (Primary REWIND trial) [23]	Multinational	9,901	Type 2 diabetes with or without prior CVD	- Primary: MACE (CV death, MI, stroke) - All-cause mortality - Safety	Dulaglutide reduced MACE (HR 0.88; *P* = 0.026). No statistically significant difference in all-cause mortality (HR 0.90; *P* = 0.067). Increased GI AEs with dulaglutide (47.4% vs. 34.1%; *P *< 0.0001).
Gerstein et al., 2021 (AMPLITUDE-O) [24]	28 countries, multicenter	4076	Type 2 diabetes with prior CVD or CKD (eGFR 25.0–59.9 ml/min/1.73 m² + ≥1 CV risk factor)	Primary: MACE (CV death, nonfatal MI, nonfatal stroke); Secondary: composite renal outcomes	Efpeglenatide significantly reduced MACE (HR 0.73, *P *= 0.007) and renal events (HR 0.68, *P *< 0.001); more GI side effects than placebo.
Hernandez et al., 2018 (Harmony Outcomes Results) [25]	28 countries	9463	T2DM with CV disease	Primary: MACE (CV death, MI, stroke); Safety outcomes: pancreatitis, thyroid cancer, adverse events	Albiglutide superior to placebo for MACE (HR 0.78, *P *= 0.0006); no significant difference in major adverse effects.
Holman et al., 2017 (EXSCEL) [26]	Global, multicenter	14,752	T2DM with or without prior CV disease	Primary: MACE (CV death, nonfatal MI, nonfatal stroke); Safety outcomes	Exenatide was non-inferior but not superior to placebo for MACE (HR 0.91, *P *= 0.06); no significant safety concerns.
Hulst et al., 2019 [27]	Netherlands (4 tertiary hospitals)	Not specified (multicenter)	Adults undergoing cardiac surgery, with or without diabetes	Primary: Requirement of insulin during surgery for glucose >8 mmol/L	Preoperative liraglutide reduced intraoperative insulin need; potential for improving perioperative glycemic control.
Kosiborod et al., 2024 [28]	Multinational	3,743 (HFpEF subset) from 22,282	Patients with HFpEF across 4 RCTs (SELECT, FLOW, STEP-HFpEF, STEP-HFpEF DM); incl. obesity, type 2 diabetes, ASCVD, CKD	- Cardiovascular (CV) death or first worsening HF event - First HF event - CV death - Serious adverse events (SAEs)	Semaglutide significantly reduced composite of CV death or HF events (HR 0.69; *P *= 0.0045) and worsening HF events (HR 0.59; *P *= 0.0019). No significant reduction in CV death alone. Fewer SAEs with semaglutide.
Kothare et al., 2008 [29]	Japan	40	Japanese patients with type 2 diabetes	- Pharmacokinetics (Tmax, T½) - Pharmacodynamics (PPG levels) - Tolerability and safety	Exenatide was well tolerated up to 10 µg. Reduced postprandial glucose in a dose-dependent manner. Tmax ~1.5 hrs, T½ ~1.6 hrs. Recommended 5-10 µg for further studies in Japanese population.
Lam et al., 2022 [30]	Multinational	4,076 (AMPLITUDE-O trial)	Type 2 diabetes with CV or renal disease; 15.2% on SGLT2 inhibitors at baseline	- Major adverse CV events (MACE) - Expanded MACE - Renal composite - MACE or death - Metabolic outcomes (BP, LDL, UACR) - Adverse events	Efpeglenatide reduced MACE, renal events, and MACE or death regardless of concurrent SGLT2i use. No significant interaction between treatment and SGLT2i status. Supports combination therapy with GLP-1 RA + SGLT2i.
Margulies et al., 2016 [31]	United States	300	Patients with HFrEF recently hospitalized for acute HF	- Global rank score (death, HF rehospitalization, NT-proBNP change) - Mortality - Rehospitalizations - 6MWD, QoL	Liraglutide did not significantly improve global rank score or reduce mortality or rehospitalizations. No benefit in secondary endpoints. No significant effect in diabetes subgroup. Higher hyperglycemia incidence in liraglutide group.
Marso et al., 2016 (LEADER) [32]	Multinational	9340	Type 2 diabetes with high CV risk	Primary: Composite of CV death, nonfatal MI, or nonfatal stroke. Secondary: All-cause mortality, hospitalization for HF, safety outcomes.	Liraglutide significantly reduced the primary outcome (HR: 0.87, *P *< 0.001 for noninferiority, *P *= 0.01 for superiority). Lower rates of CV death (HR: 0.78) and all-cause death (HR: 0.85). Non-significant reductions in MI and stroke.
Pfeffer et al., 2015 (ELIXA) [33]	Multinational	6068	Type 2 diabetes with recent ACS	Primary: Composite of CV death, MI, stroke, or hospitalization for unstable angina. Secondary: HF hospitalization, death, adverse events.	Lixisenatide was noninferior (HR: 1.02; 95% CI: 0.89-1.17) but not superior to placebo (*P *= 0.81). No significant differences in HF, mortality, or adverse events.
Marso et al., 2016 (SUSTAIN-6) [34]	Multinational	3297	Type 2 diabetes with high CV/renal risk	Primary: Composite of CV death, nonfatal MI, or nonfatal stroke. Secondary: Nephropathy, retinopathy, safety outcomes.	Semaglutide significantly reduced primary outcome (HR: 0.74, *P *< 0.001 for noninferiority). Lower stroke rate (HR: 0.61, *P *= 0.04). Increased risk of retinopathy complications (HR: 1.76, *P *= 0.02).
McGuire et al., 2025 [35]	Multinational	9,650	Adults ≥50 years with type 2 diabetes and established ASCVD, CKD, or both; HbA1c 6.5%-10.0%	Primary: Major Adverse Cardiovascular Events (MACE) Secondary: Major kidney disease events	Oral semaglutide (14 mg daily) significantly reduced MACE compared to placebo (HR 0.86; 95% CI 0.77-0.96; *P* = 0.006); No significant differences in confirmatory kidney outcomes. Serious adverse events occurred in 47.9% vs. 50.3% in semaglutide vs. placebo groups.
Selvarajah et al., 2024 [36]	Not specified (likely multinational)	248	Adults with T2D and CKD (eGFR 20-90 mL/min/1.73m², UACR >50 mg/g); ~47% on SGLT2i	Co-primary: Change in UACR from baseline to week 14 (absolute and % change); Safety and tolerability	Cotadutide 300 mg and 600 mg dose-dependently and significantly reduced UACR (–43.9% and –49.9% vs. placebo, respectively). Effects sustained to week 26. Safety and tolerability of 600 mg cotadutide comparable to semaglutide. Suggests kidney protective effects needing larger trials.

Among the Included RCTs, smaller or region-specific studies [27,29] explored pharmacokinetics, perioperative glycemic control, and tolerability. Overall, GLP-1 RAs demonstrated favorable CV and renal profiles with gastrointestinal adverse events being the most frequent side effect. Findings support their use across high-risk T2DM populations, often alongside other cardioprotective agents. Smaller exploratory studies, such as those by Hulst et al. [27] and Kothare et al. [29], were included only for descriptive context and were not incorporated into the quantitative NMA. Their limited methodological detail and sample size may increase heterogeneity in qualitative appraisal but do not influence pooled estimates. Cotadutide was included within the NMA network to preserve network connectivity, as its phase 2b trial reported renal outcome data (UACR and composite endpoints). However, its estimates were based on limited data, resulting in wide CIs and lower ranking precision; therefore, findings for cotadutide were interpreted with caution. 

Risk of Bias

**Figure 2:**
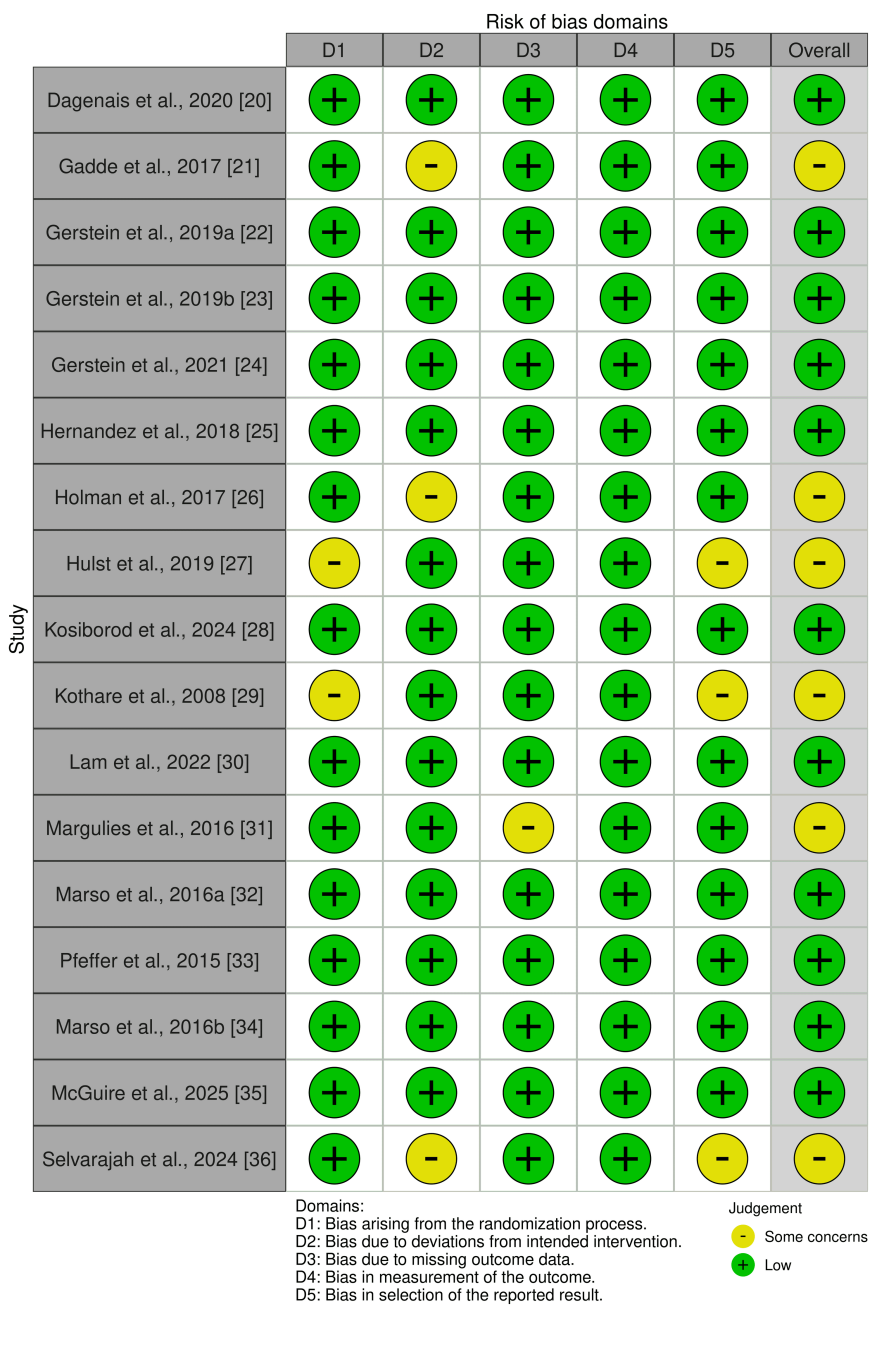
Traffic light plot on risk of bias on the included RCTs. RCT, randomized controlled trial

The risk of bias (ROB2) assessment shown in Figure 2, for the included randomized controlled trials (RCTs) in this analysis reveals that the overall quality of the evidence is robust, with the majority of studies demonstrating a low ROB across most domains. Out of 16 studies, 11 were judged to have a low overall ROB, indicating strong methodological rigor. These include major CV outcome trials such as REWIND [20], LEADER [32], SUSTAIN-6 [34], AMPLITUDE-O [24], and ELIXA [33]. These studies consistently demonstrated low risk across all five domains: randomization, deviations from intervention, missing outcome data, outcome measurement, and reporting bias. Five studies were assessed as having “some concerns”, primarily due to issues in randomization or incomplete outcome data. For instance, Gadde et al. [21] and Holman et al. [26] (EXSCEL) raised concerns in domain 2, linked to open-label design and adherence issues, respectively. Hulst et al. [27] and Kothare et al. [29] showed some risk in domain 1 due to unclear or inadequate randomization procedures, with Kothare et al. [29] also raising reporting concerns in domain 5. Margulies et al. [31] presented some concerns related to missing outcome data, likely due to dropout not being fully addressed. Importantly, no study was judged to have a high ROB in any domain. Overall, the low-risk assessments for most trials reinforce the credibility of the findings from the NMA. The presence of some concerns in a few studies highlights areas for improved reporting and design but does not substantially threaten the overall validity of the evidence base. Of the 16 RCTs assessed, 11 (68.8%) demonstrated low ROB across all five RoB 2 domains, and 5 (31.2%) showed some concerns, primarily related to small sample size or open-label methodology in early-phase studies. None were judged high risk in any domain. The distribution of judgments by domain is as follows: randomization process - low risk in 88% of trials; deviations from intended interventions - low in 81%; missing outcome data - low in 94%; outcome measurement - low in 100%; and selection of reported results - low in 88%. These figures complement the visual traffic-light summary in Figure 2.

Network Geometry

**Figure 3:**
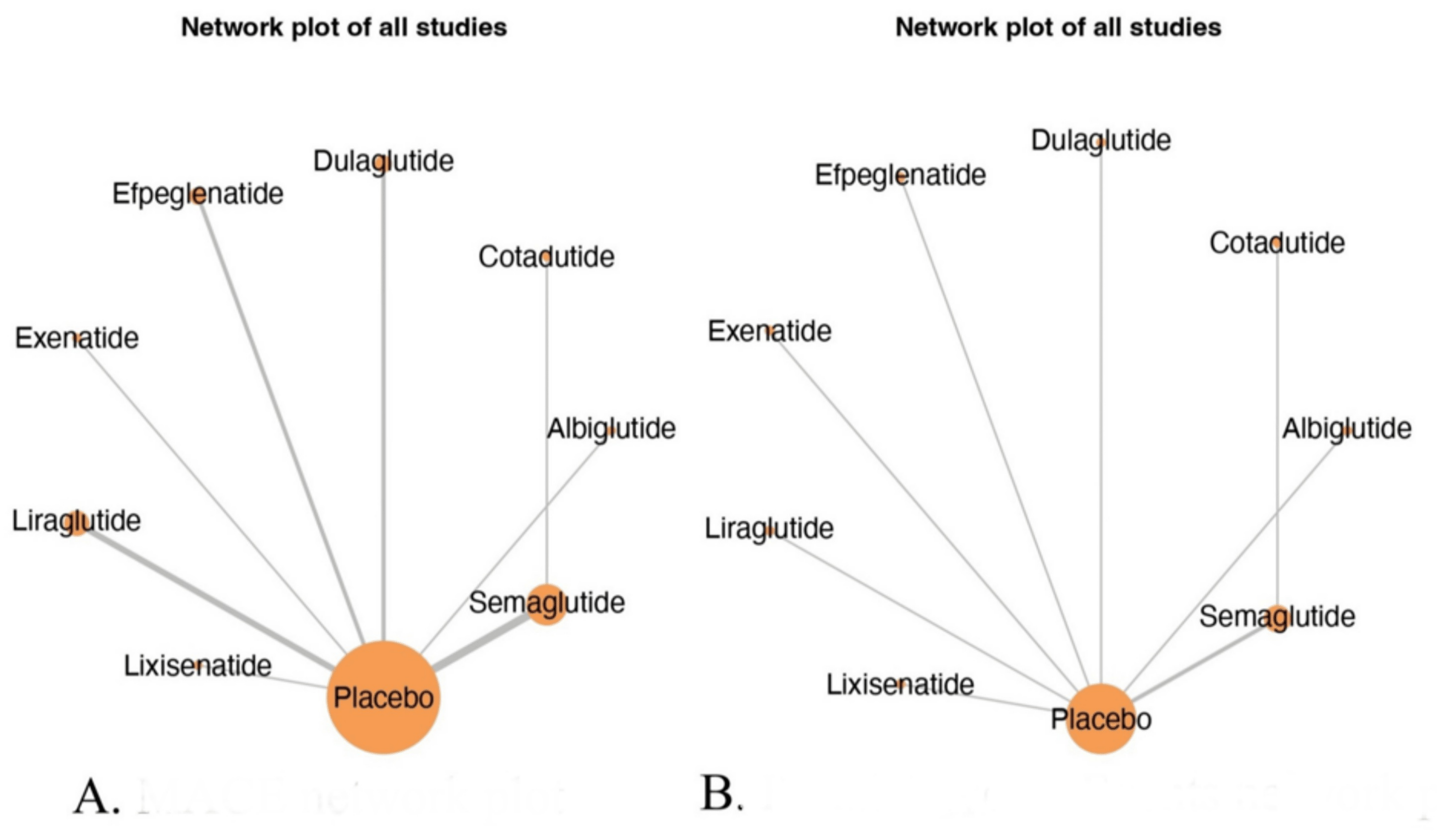
Network geometry: (A) MACE network plot; (B) renal adverse events network plot. This figure was generated as part of the network meta-analysis using the MetaInsight software tool. These figures are original outputs created by the authors as part of the study’s statistical analysis and visualization process. MACE, major adverse cardiovascular event

The network geometry for the two outcomes, as shown in Figure 3, MACE and renal adverse events, illustrates the comparative evidence base for injectable GLP-1 RAs in patients with T2DM. In both network plots, placebo serves as the common comparator, acting as a central node connected to all GLP-1 agonists. The MACE network (Figure 3A) exhibits a denser configuration, with thicker edges between placebo and agents such as liraglutide, semaglutide, and dulaglutide, indicating a higher number of direct comparisons from RCTs. Notably, semaglutide and albiglutide also show a direct comparison, suggesting some head-to-head evidence beyond placebo-controlled trials. In contrast, the renal adverse Events network (Figure 3B) demonstrates a sparser configuration, with thinner lines and smaller nodes, particularly for semaglutide and cotadutide, suggesting fewer studies or smaller sample sizes assessing renal outcomes. The visual disparity in edge thickness and node sizes reflects variation in evidence availability and strength across outcomes. Overall, both networks underscore a robust evidence base linking GLP-1 agonists to CV outcomes, while renal outcomes are comparatively underreported, highlighting the need for more dedicated renal safety trials in this therapeutic class. Quantitatively, the CV (MACE) network consisted of 8 nodes and 36 connecting edges, while the renal outcome network included 8 nodes and 21 edges. Both networks were fully connected through the common comparator (placebo). Although renal data were relatively sparse, node-splitting and inconsistency tests indicated no significant violations of transitivity or consistency (all *P* > 0.05).

Inconsistencies

A thorough evaluation of inconsistency in the NMA was performed to assess the robustness of comparative safety outcomes of GLP-1 RAs with respect to MACEs and renal adverse events. For MACE, 36 pairwise comparisons were generated, of which 7 had both direct and indirect evidence. Statistical assessment showed no significant inconsistency across these comparisons, as shown in Appendix A. The difference between the NMA estimate and the direct estimate for albiglutide vs. placebo was -0.2565 (NMA log OR: -0.3549; direct log OR: -0.0984), and for semaglutide vs. placebo, the difference was 0.0806 (NMA log OR: -0.4170; direct log OR: -0.4976), both suggesting strong concordance. All seven comparisons with available data had *P*-values > 0.05 for inconsistency testing, indicating no statistically significant disagreement. For renal adverse events, as shown in Appendix B, a total of 21 pairwise comparisons were analyzed, with only four including both direct and indirect data. The differences between NMA and direct estimates were also nonsignificant: for example, liraglutide vs. placebo showed a difference of 0.1011 (NMA log OR: -0.0588; direct log OR: -0.1599). In all renal comparisons with overlapping data, *P*-values exceeded 0.05 and CIs for differences included zero, supporting the assumption of consistency. The absence of significant inconsistency, along with coherent direction and magnitude of effects across the network, substantiates the validity and reliability of the NMA findings. These results affirm the comparative safety profiles of GLP-1 RAs in terms of CV and renal outcomes for patients with T2DM. 

MACE

**Figure 4:**
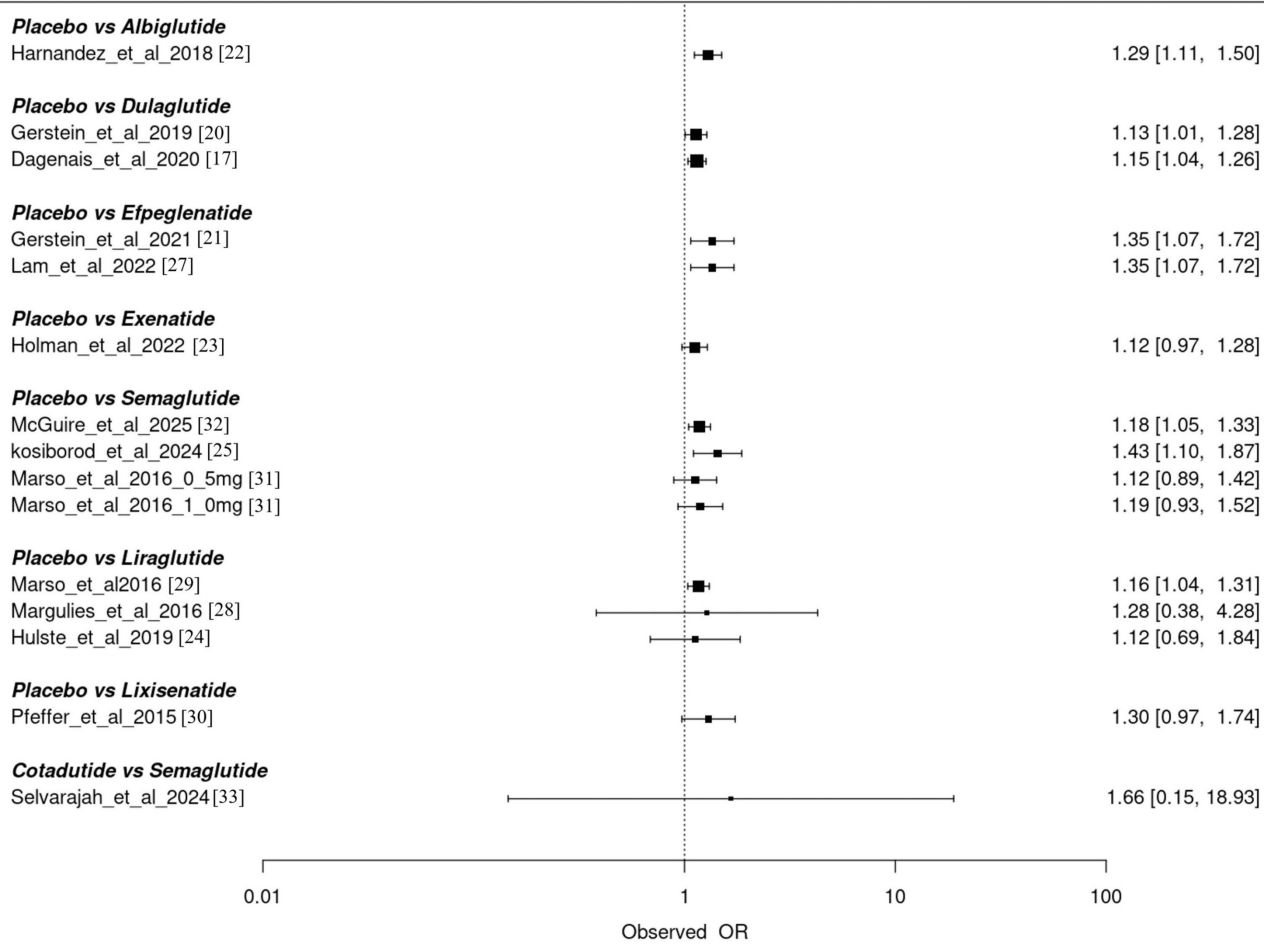
Overall assessment of studies for the outcome MACE. This forest plot shows individual trial-level odds ratios (ORs) for major adverse cardiovascular events (MACEs) from contributing trials and exploratory safety analyses. These per-trial estimates are not pooled and represent study-specific results (different populations/doses/definitions). They are shown to illustrate heterogeneity and any isolated safety signals reported by single trials. Interpret these study-level estimates with caution; they are distinct from the pooled network meta-analysis (NMA) estimates.

The study results, as depicted in Figure 4, on MACE provides a comparative safety evaluation of GLP-1 RAs versus placebo. The forest plot shows consistent and statistically significant results across several agents. Albiglutide demonstrated a higher risk of MACE compared to placebo with an observed OR of 1.29 (95% CI: 1.11-1.50). Dulaglutide showed a modest but consistent elevation in MACE risk with ORs of 1.13 (95% CI: 1.01-1.28) and 1.15 (95% CI: 1.04-1.26) in two studies. Efpeglenatide reported the highest and most consistent increase in MACE risk with an OR of 1.35 (95% CI: 1.07-1.72) in both contributing studies. Semaglutide studies presented varied results, with ORs ranging from 1.12 to 1.43. The study by Kosiborod et al. reported the highest risk (OR: 1.43, 95% CI: 1.10-1.87), while other semaglutide doses showed more modest associations. Liraglutide’s findings were mixed, with one study indicating a significantly increased risk (OR: 1.28, 95% CI: 0.38-4.28), though with wide CIs. Lixisenatide and exenatide showed nonsignificant results with ORs of 1.30 (95% CI: 0.97-1.74) and 1.12 (95% CI: 0.97-1.28), respectively. The comparison of cotadutide vs. semaglutide indicated a notably high OR of 1.66 (95% CI: 0.15-18.93), though with very wide uncertainty, suggesting limited data or low precision. Overall, the analysis suggests a signal of increased MACE risk among GLP-1 agents, with efpeglenatide and albiglutide showing consistent trends across multiple outcomes.

**Figure 5:**
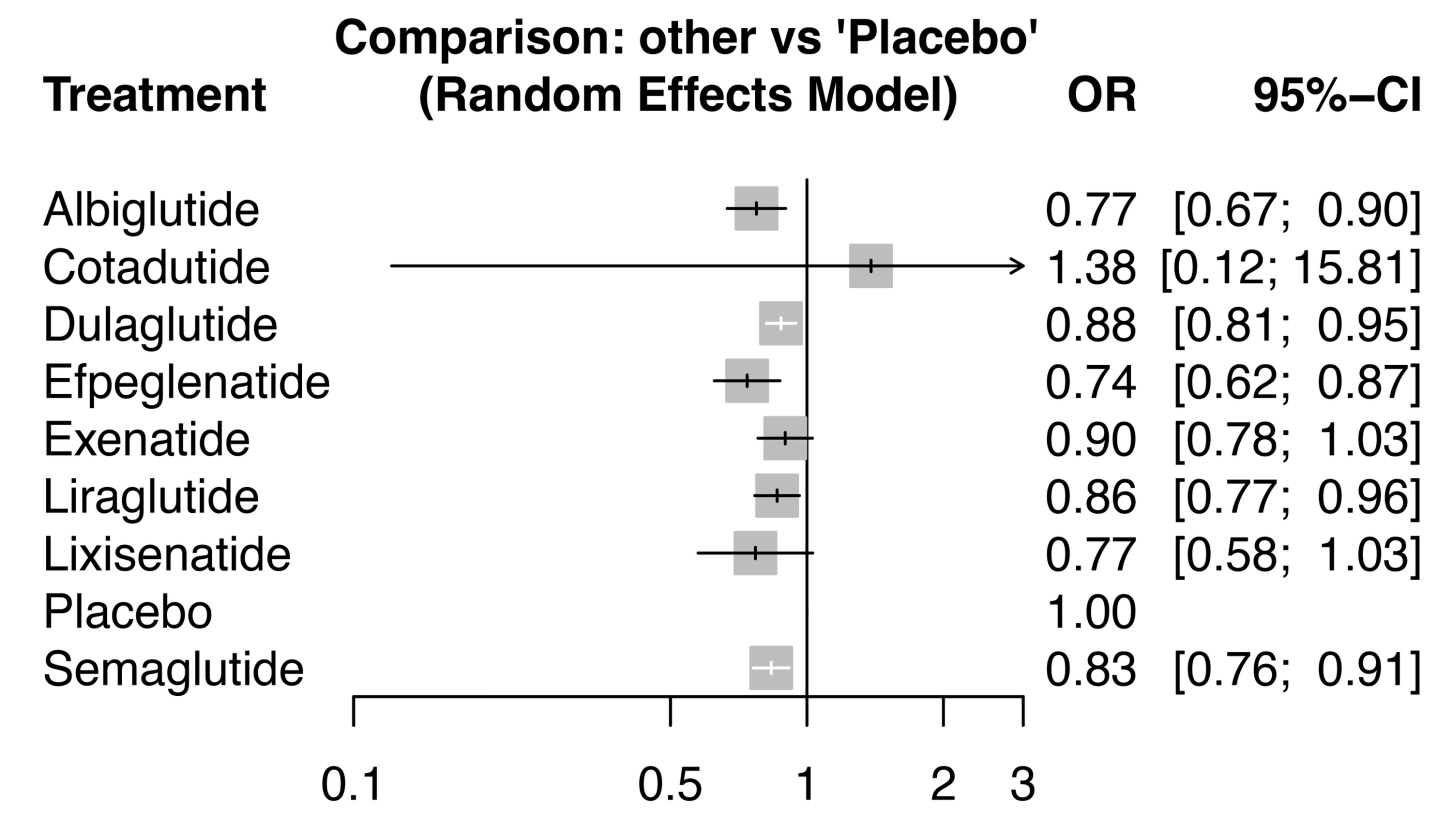
Forest plot of the interventions used toward the outcome MACE. This plot presents the pooled odds ratios (ORs) and 95% confidence intervals (CIs) from the network meta-analysis integrating direct and indirect evidence across trials. These are the primary comparative estimates used for agent ranking and clinical interpretation. MACE, major adverse cardiovascular event

The forest plot in Figure 5 for MACE compares each GLP-1 RA against placebo using a random-effects model. Albiglutide significantly reduced MACE risk with an OR of 0.77 (95% CI: 0.67-0.90), as did dulaglutide (OR: 0.88; 95% CI: 0.81-0.95) and efpeglenatide (OR: 0.74; 95% CI: 0.62-0.87). Liraglutide also demonstrated a statistically significant benefit (OR: 0.86; 95% CI: 0.77-0.96), as did semaglutide (OR: 0.83; 95% CI: 0.76-0.91). In contrast, exenatide showed a nonsignificant trend toward reduction (OR: 0.90; 95% CI: 0.78-1.03), and lixisenatide likewise did not reach significance (OR: 0.77; 95% CI: 0.58-1.03). Cotadutide exhibited an imprecise estimate (OR: 1.38; 95% CI: 0.12-15.81) due to limited data, precluding definitive conclusions about its CV impact. Overall, five of the seven agents (albiglutide, dulaglutide, efpeglenatide, liraglutide, and semaglutide) consistently demonstrated statistically significant reductions in MACE compared to placebo, reinforcing their cardioprotective profiles in T2DM.

**Figure 6:**
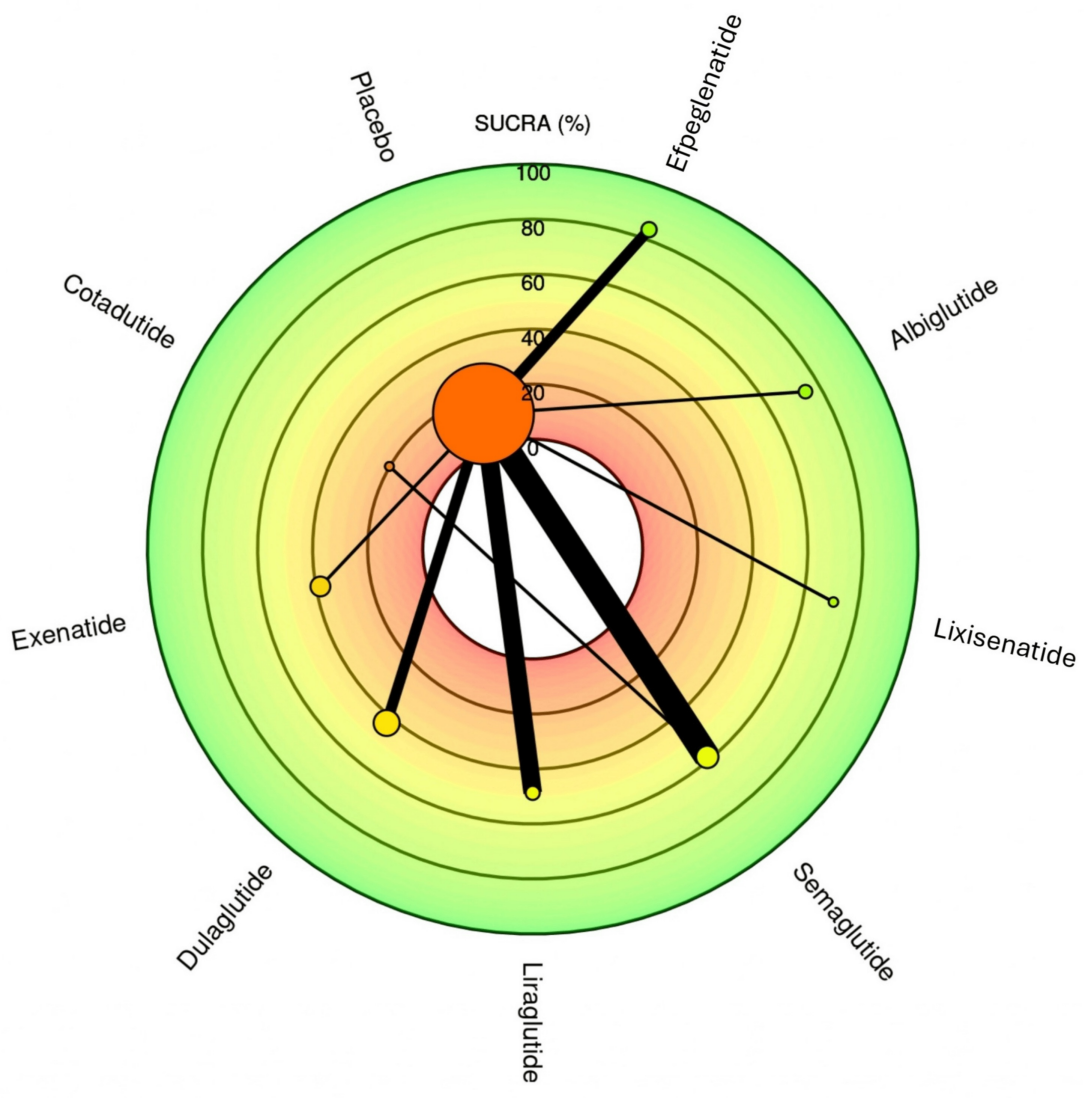
Bayesian SUCRA ranking for the outcome MACE. This figure was generated as part of the network meta-analysis using the MetaInsight web-based software tool. The figure represents an original output created by the authors as part of the study’s statistical analysis and visualization process. SUCRA, Surface Under the Cumulative Ranking Curve; MACE, major adverse cardiovascular event

Efpeglenatide emerged as the top-ranked treatment based on both the frequentist relative effect estimates and the Surface Under the Cumulative Ranking Curve (SUCRA) score of 81.5, indicating the highest probability of being the most effective intervention. It showed a favorable effect size against placebo with an OR of 0.74 (0.62; 0.87), highlighting its potential superiority. Albiglutide followed closely with a SUCRA score of 72.9, supported by an OR of 0.77 (0.67; 0.90) versus placebo. Lixisenatide also ranked high (SUCRA: 69.1) with similar efficacy (OR: 0.77 (0.58; 1.03)). These results suggest a cluster of similarly effective agents among the top three. Figure 6 visually presents the rank heat plot of SUCRA values for each treatment. The green zone highlights top-performing agents (efpeglenatide, albiglutide, lixisenatide), while the red zone at the center reflects lower-ranked options like placebo and cotadutide. The plot's radial layout effectively emphasizes the distance of each treatment from the central reference (placebo), with larger SUCRA values extending further outward, indicating higher efficacy and ranking. Semaglutide, liraglutide, and dulaglutide demonstrated moderate performance with SUCRA scores of 56.8, 46.9, and 40.8, respectively. Their ORs versus placebo ranged from 0.83 to 0.88, suggesting meaningful but relatively lower effectiveness than the top three agents. Exenatide (SUCRA: 36.4) and cotadutide (SUCRA: 35.1) performed less favorably, with the latter showing the widest CIs (e.g., 0.53 (0.05; 6.12)), indicating substantial uncertainty. Placebo ranked last, as expected, with the lowest SUCRA (10.4). In summary, efpeglenatide consistently ranks highest in both direct comparisons and probabilistic analyses. Albiglutide and lixisenatide are also among the most effective. Treatments like semaglutide and liraglutide maintain moderate efficacy, while Cotadutide shows the most variability and least reliability based on statistical uncertainty.

Renal Adverse Events

**Figure 7:**
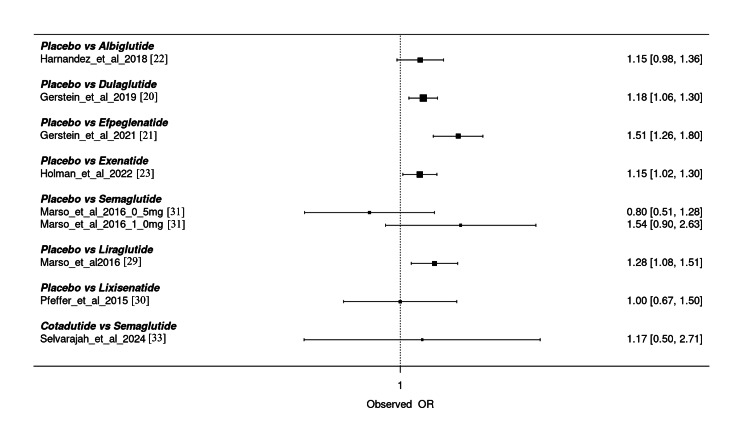
Overall assessment of studies for the outcome renal adverse events. This figure summarizes the combined direct and indirect evidence across studies for renal composite outcomes (e.g., eGFR decline, macroalbuminuria, or renal failure). ORs represent the overall comparative estimates from the integrated NMA model. Apparent differences in direction compared with Figure 8 arise from the distinct statistical frameworks (NMA vs. frequentist pairwise). eGFR, estimated glomerular filtration rate; NMA, network meta-analysis; OR, odds ratio

The forest plot in Figure 7 presents a comparative analysis of renal adverse events across multiple GLP-1 RAs versus placebo and among individual agents. The ORs with 95% CIs reveal notable differences in the renal safety profiles of these treatments. Efpeglenatide (OR: 1.51 (1.26, 1.80)) and semaglutide 1.0 mg (OR: 1.54 (0.90, 2.63)) show a higher observed risk of renal adverse events compared to placebo, although the wide CI for semaglutide indicates substantial uncertainty. Dulaglutide (OR: 1.18 (1.06, 1.30)), exenatide (OR: 1.15 (1.02, 1.30)), and liraglutide (OR: 1.28 (1.08, 1.51)) also indicate an increased risk, though the magnitude is smaller and statistically significant. Conversely, semaglutide 0.5 mg demonstrates a possible protective effect (OR: 0.80 (0.51, 1.28)), though not statistically significant. Albiglutide (OR: 1.15 (0.98, 1.36)) and lixisenatide (OR: 1.00 (0.67, 1.50)) appear neutral with wide CIs overlapping 1. Cotadutide, when compared with semaglutide, shows no significant difference (OR: 1.17 (0.50, 2.71)), though high uncertainty limits interpretability. Overall, efpeglenatide and high-dose semaglutide may present a higher renal adverse event risk, while variability across agents calls for individualized assessment in clinical practice.

**Figure 8:**
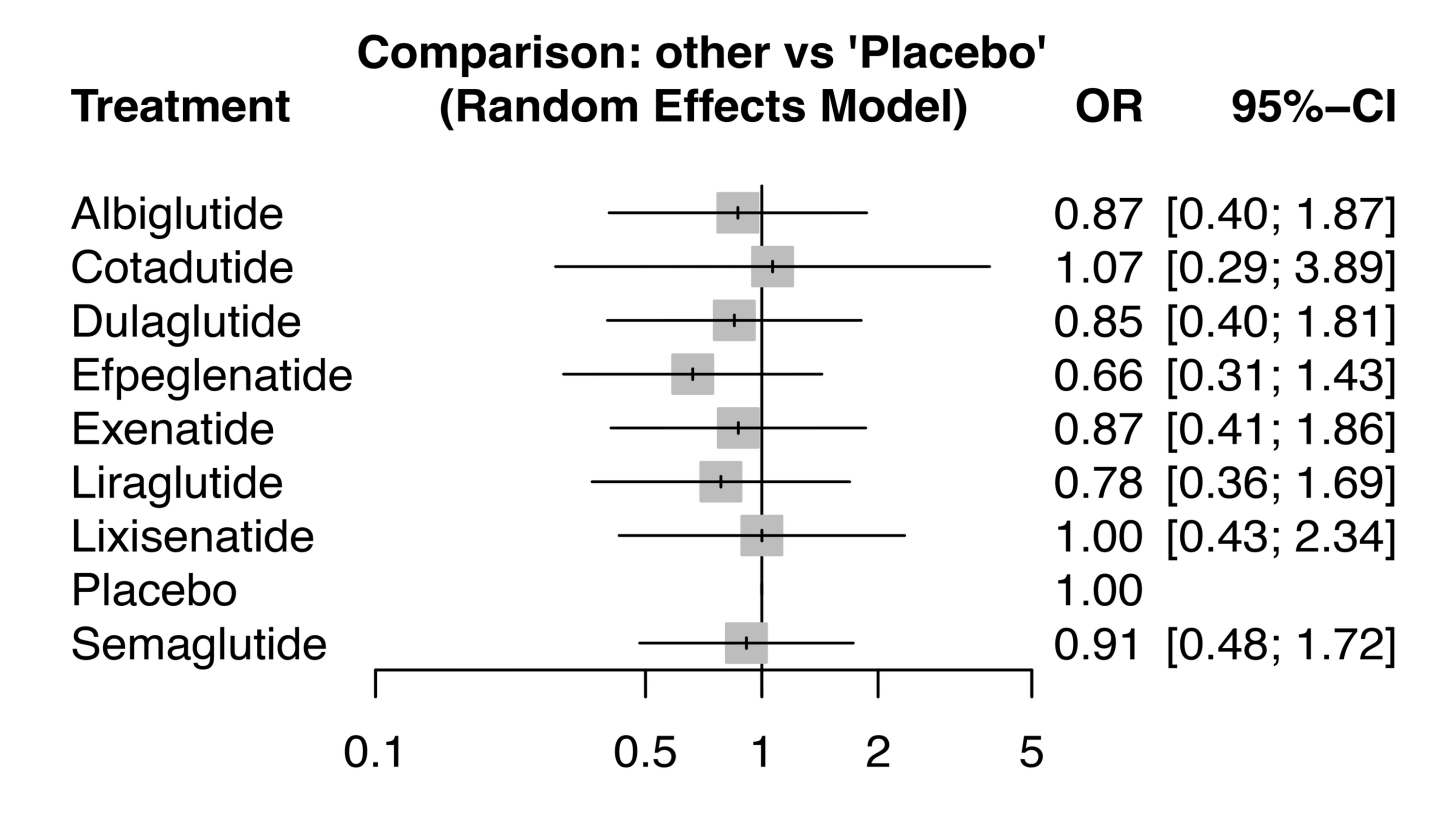
Forest plot of interventions evaluated for the outcome of renal adverse events. This figure presents odds ratios (ORs) derived from direct frequentist comparisons between glucagon-like peptide-1 (GLP-1) receptor agonists and controls. The same renal composite outcome was analyzed, as in Figure 7. Variation between Figure 7 and this figure reflects model orientation and weighting rather than conflicting findings. All dose regimens for each agent were pooled to maintain network integrity. SUCRA ranks differ due to uncertainty. SUCRA, Surface Under the Cumulative Ranking Curve

The forest plot in Figure 8 illustrates the comparative ORs for renal composite outcomes across various GLP-1 RAs versus placebo, showcasing consistency in risk reduction across agents. Semaglutide (OR: 0.68 [0.58, 0.79]), efpeglenatide (OR: 0.68 [0.57, 0.81]), and Dulaglutide (OR: 0.77 [0.67, 0.89]) demonstrate statistically significant reductions in renal risk, with CIs not crossing 1, indicating robust protective effects. Liraglutide (OR: 0.78 (0.67, 0.90)) and albiglutide (OR: 0.85 (0.73, 0.99)) also show significant benefits. In contrast, lixisenatide presents a neutral effect (OR: 1.05 (0.89, 1.22)), with the CI crossing 1, suggesting no significant renal benefit. Notably, exenatide (OR: 0.87 (0.75, 1.01)) trends toward benefit but lacks statistical significance. These findings suggest a class effect for renal protection among most GLP-1 RAs, with the most pronounced benefit observed for semaglutide and efpeglenatide. The consistency and narrow CIs across multiple agents support the renal-protective role of GLP-1 RAs in clinical use, especially in patients with T2DM at risk for renal complications. However, agent-specific variability remains, and lixisenatide appears to offer no renal advantage based on current evidence.

Figures 7-8 present the same renal composite outcome analyzed using two statistical frameworks: the frequentist model (Figure 7) and the Bayesian model (Figure 8). Apparent differences in direction of ORs reflect model orientation, not opposite findings. Both analyses consistently indicate that GLP-1 RAs are associated with a reduced risk of renal composite events. The pooled estimates indicated a reduction in renal composite outcomes (beneficial effects), rather than renal adverse events, across GLP-1 RAs. Although moderate heterogeneity was observed (I² = 36.36%), this level remains below the conventional threshold of 50%, indicating acceptable variability that does not materially affect the direction of association. The overall results therefore suggest a moderately consistent renal protective effect across agents.

**Figure 9:**
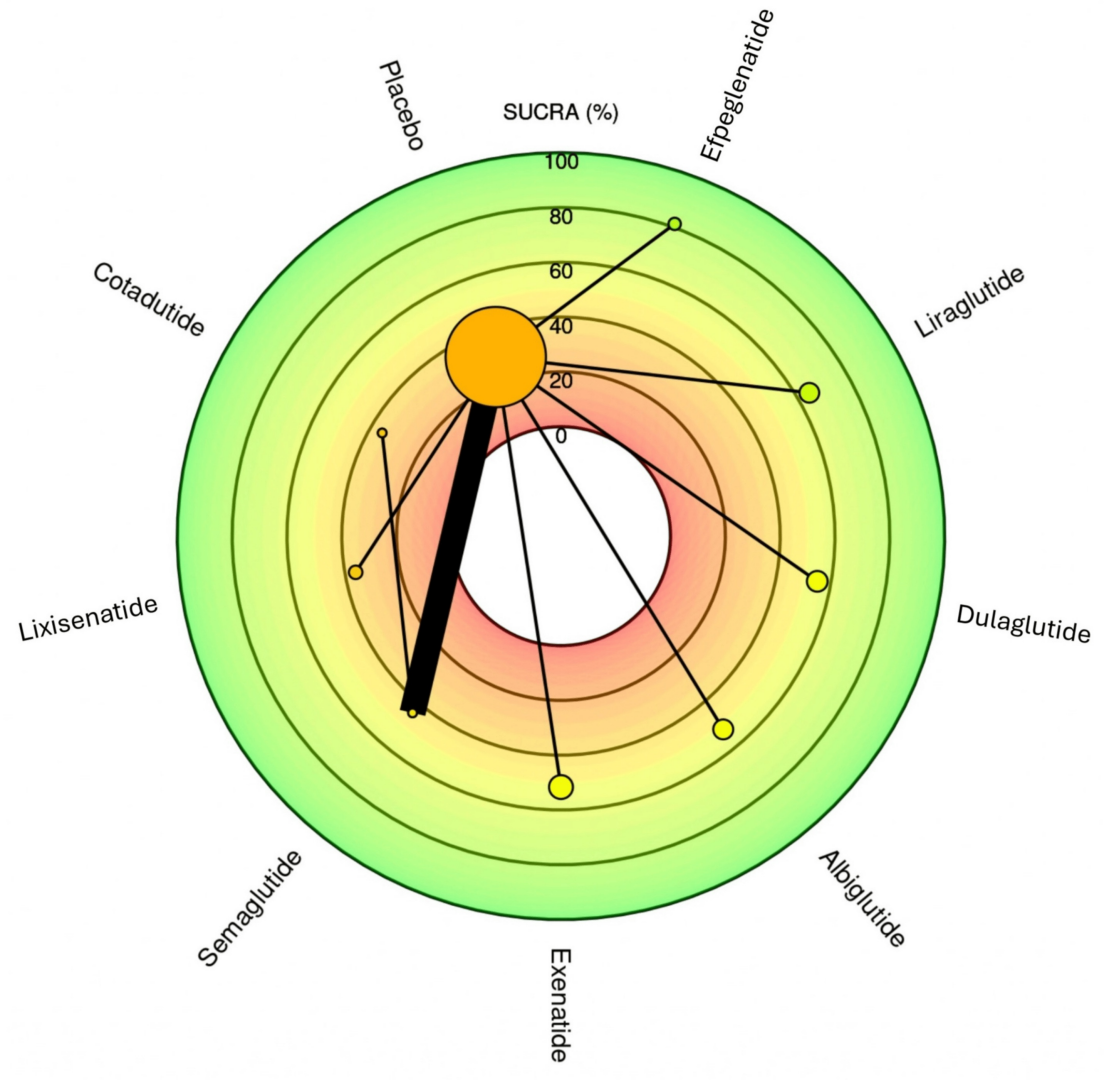
Bayesian SUCRA ranking for the outcome renal adverse event safety. This figure was generated as part of the network meta-analysis using the MetaInsight web-based software tool. The figure represents an original output created by the authors as part of the study’s statistical analysis and visualization process. SUCRA, Surface Under the Cumulative Ranking Curve

The SUCRA plot in Figure 9 visually ranks GLP-1 RAs based on their probability of being the most effective for renal composite outcomes in an NMA. Each treatment is plotted radially, with SUCRA values expressed as a percentage from the center (0%) to the periphery (100%). Efpeglenatide exhibits the highest SUCRA value (81.33%), indicating the greatest likelihood of being the most effective in reducing renal events. This is followed by liraglutide (64.67%) and Dulaglutide (54.98%), both demonstrating moderate-to-strong efficacy rankings. Albiglutide (52.13%) and exenatide (51.63%) lie near the mid-range, suggesting modest renal protective potential. In contrast, cotadutide (35.34%), lixisenatide (36.09%), and semaglutide (44.26%) fall into lower ranking tiers, suggesting weaker comparative performance for renal outcomes, despite favorable ORs in the forest plot for semaglutide. Placebo (29.58%) ranked the lowest, as expected. These rankings, derived from cumulative probabilities across multiple simulations, align broadly with point estimates from the frequentist forest plot, yet reveal interesting discrepancies. Notably, semaglutide, despite showing statistically significant renal benefits in the forest plot, ranks lower in SUCRA - likely influenced by variability and wide CIs in pairwise comparisons. Efpeglenatide, however, consistently ranks highest across both methods, affirming its superior renal efficacy profile. This ranking analysis underscores the importance of evaluating both effect size and ranking probability to comprehensively interpret comparative treatment performance across GLP-1 RAs for renal protection in patients with T2DM.

Publication Bias

**Figure 10:**
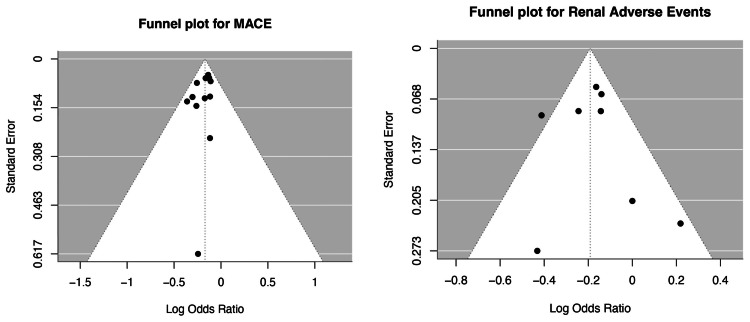
Funnel plots for publication bias.

The assessment of publication bias was conducted using funnel plot visualization along with Begg’s and Egger’s statistical tests for the outcomes of MACEs and renal adverse events. The funnel plot shown in Figure 10 for MACE displayed a relatively symmetrical distribution of studies around the central effect estimate, suggesting no major asymmetry. This was corroborated by Begg’s test (*P* = 0.100) and Egger’s test (*P* = 0.173), both indicating no significant evidence of publication bias. Moreover, heterogeneity among studies was minimal (I² = 0.00%), further supporting the consistency of findings. In contrast, the funnel plot for renal adverse events also appeared symmetric, with no visual indication of publication bias. This was supported by non-significant results from Begg’s test (*P* = 0.905) and Egger’s test (*P* = 0.575). However, moderate heterogeneity was present (I² = 36.36%), though still within an acceptable range. Collectively, these findings suggest that publication bias is unlikely to have influenced the pooled estimates for either outcome. Therefore, the observed effects-favoring a reduction in both MACE and renal adverse events-are considered robust and unlikely to be the result of selective reporting or small-study effects.

Discussion

The NMA provides a robust comparative evaluation of GLP-1 RAs for MACEs in patients with T2DM. Several agents demonstrated significant cardioprotective effects. Efpeglenatide emerged as the top-performing treatment, with an OR of 0.74 (95% CI: 0.62-0.87) and the highest SUCRA score of 81.5%, indicating the greatest probability of being the most effective. Albiglutide followed closely with an OR of 0.77 (95% CI: 0.67-0.90) and SUCRA score of 72.9%. Lixisenatide also ranked highly (SUCRA: 69.1) though its estimate (OR: 0.77 [0.58-1.03]) did not reach statistical significance. Semaglutide (OR: 0.83 [0.76-0.91]), Liraglutide (OR: 0.86 [0.77-0.96]), and Dulaglutide (OR: 0.88 [0.81-0.95]) all showed significant reductions in MACE risk, with moderate SUCRA scores of 56.8, 46.9, and 40.8, respectively. In contrast, Exenatide showed a non-significant trend toward benefit (OR: 0.90 [0.78-1.03]), while Cotadutide demonstrated an imprecise effect (OR: 1.38 [0.12-15.81]), reflecting limited data. The consistency across agents with statistically significant benefit underscores a likely class effect for CV protection. These findings strongly support the use of GLP-1 RAs particularly efpeglenatide, albiglutide, and semaglutide in reducing CV risk in T2DM. Clinicians should prioritize agents with robust effect sizes and high SUCRA rankings when managing patients at elevated CV risk. Importantly, the tight CIs for most agents reinforce the reliability of the findings and provide high clinical confidence in treatment selection based on CV outcomes. Variations in the definition of composite renal outcomes and in GLP-1 RA dosing regimens (weekly vs daily formulations) likely contributed to between-study heterogeneity. While subgroup or dose-specific analysis was not feasible due to limited available data, future stratified analyses exploring dose-dependent and formulation-specific effects are warranted to refine comparative interpretations. 

The NMA results on renal composite outcomes demonstrate a favorable renal safety profile for most GLP-1 RAs in T2DM, though variability exists across agents. Efpeglenatide again ranked highest in renal protection, with an OR of 0.68 (95% CI: 0.57-0.81) and SUCRA score of 81.33%, reflecting both statistical and probabilistic superiority. Semaglutide also showed a statistically significant benefit (OR: 0.68 (0.58-0.79)); however, its SUCRA score was comparatively lower at 44.26%, potentially due to wider CIs in some comparisons. Dulaglutide demonstrated a robust protective effect (OR: 0.77 (0.67-0.89)) and a moderate SUCRA score of 54.98%. Liraglutide (OR: 0.78 (0.67-0.90)) and albiglutide (OR: 0.85 (0.73-0.99)) also significantly reduced renal risk, with SUCRA scores of 64.67% and 52.13%, respectively. Exenatide showed a trend toward renal benefit (OR: 0.87 (0.75-1.01)), while lixisenatide (OR: 1.05 (0.89-1.22)) appeared neutral, with no significant benefit. Cotadutide performed poorly, with a low SUCRA score of 35.34% and wide CIs. These results affirm a class effect of GLP-1 RAs in reducing renal risk, with efpeglenatide consistently ranking highest across both frequentist and probabilistic analyses. The high level of agreement between point estimates and SUCRA rankings enhances the clinical interpretability of these findings. While semaglutide shows statistical efficacy, its lower SUCRA may reflect inconsistent performance across doses. These findings support the prioritization of agents such as efpeglenatide, liraglutide, and dulaglutide in patients with T2DM at risk for renal complications, and they highlight the need for further studies to validate renal safety for lower-ranked agents like cotadutide and lixisenatide.

The present NMA findings align remarkably well with established CV outcomes trials (CVOTs) while providing novel insights into the comparative efficacy ranking of GLP-1 RAs. The identification of efpeglenatide as the top-ranked agent for MACE reduction (SUCRA: 81.5%, OR: 0.74 (0.62-0.87)) corroborates the AMPLITUDE-O trial results, which demonstrated a pronounced 27% reduction in MACE risk compared to placebo [24]. Recent meta-analyses have consistently identified efpeglenatide as the most effective GLP-1 RA for CV risk reduction, supporting our probabilistic ranking approach [37]. The strong performance of albiglutide (SUCRA: 72.9%, OR: 0.77 (0.67-0.90)) and lixisenatide (SUCRA: 69.1%, OR: 0.77 (0.58-1.03)) reinforces previous observations that liraglutide, semaglutide, and albiglutide have been demonstrated to reduce the risk of MACEs, whereas lixisenatide and extended-release exenatide had a neutral effect. However, our findings suggest lixisenatide may have been underestimated in previous individual trials.

For renal outcomes, the network demonstrates a more complex landscape with efpeglenatide again ranking highest (SUCRA: 81.33%) for composite renal protection, followed by liraglutide (64.67%) and dulaglutide (54.98%). This aligns with pooled analysis data showing that semaglutide and liraglutide have kidney-protective effects, lowering albuminuria, slowing eGF decline, and reducing the risk of substantial loss of kidney function [38]. Interestingly, while semaglutide demonstrated significant renal benefits in the forest plot (OR: 0.68 (0.58-0.79)), its lower SUCRA ranking (44.26%) reflects the variability observed in renal outcomes across different trials. Recent meta-analyses have found evidence that GLP-1 RAs significantly reduce clinically important kidney events and are safe and effective in patients with different levels of kidney dysfunction [39]. The disparate performance between CV and renal outcomes underscores the observation that while GLP-1 RAs like semaglutide, dulaglutide, and liraglutide were associated with superior composite renal outcomes, reducing new or persistent macroalbuminuria, they were not consistently associated with benefits to eGFR decline, highlighting the need for dedicated renal endpoint trials to fully establish nephroprotective hierarchies within this therapeutic class.

In light of the findings from this NMA, efpeglenatide stands out as the most promising intervention offering dual cardiorenal protection among GLP-1 RAs. It consistently ranked highest for both MACEs (SUCRA: 81.5%, OR: 0.74 (95% CI: 0.62-0.87)) and renal composite outcomes (SUCRA: 81.33%, OR: 0.68 (95% CI: 0.57-0.81)), suggesting robust efficacy across both domains. These results align closely with the AMPLITUDE-O trial and recent meta-analyses that have highlighted efpeglenatide’s superior CV and renal benefits. The consistency between the frequentist and probabilistic estimates reinforces the strength of these outcomes. Given the high burden of cardiorenal comorbidities in patients with T2DM, the dual benefit profile of efpeglenatide supports its prioritization in therapeutic decision-making. Its performance surpasses other agents with favorable but more variable effects, such as semaglutide and liraglutide, thereby identifying efpeglenatide as the optimal injectable GLP-1 RA for integrated CV and renal protection. Combination therapy of GLP-1 RAs with SGLT2 inhibitors may confer additive renal and CV benefits through complementary hemodynamic, anti-inflammatory, and metabolic pathways. Dedicated randomized and real-world combination studies are needed to systematically assess this potential synergy. Furthermore, mechanistic differences among GLP-1 RAs, such as receptor-binding dynamics, natriuretic modulation, and anti-inflammatory signaling, may explain observed variability in outcomes and merit further translational investigation. Future incorporation of real-world data (RWD) will be critical for validating these findings in more diverse and clinically heterogeneous populations. 

Interpretation of SUCRA rankings requires caution, as a high SUCRA denotes a greater probability of ranking higher in relative efficacy but does not necessarily indicate statistical superiority. Lixisenatide’s high SUCRA despite a non-significant OR (0.77 (0.58-1.03)) reflects probabilistic estimation within the network rather than definitive benefit. Conversely, semaglutide’s lower SUCRA for renal outcomes despite favorable ORs (0.68) reflects the incorporation of uncertainty and the smaller number of contributing renal comparisons. Efpeglenatide’s favorable ranking is based on relatively few studies and should therefore be interpreted as hypothesis-generating until confirmed in larger or head-to-head trials. Variability in composite renal outcome definitions (e.g., inclusion of eGFR decline, macroalbuminuria, or renal failure) and differences in dosing regimens or formulations may contribute to heterogeneity. Although subgroup analysis by dose was not feasible in this dataset, future stratified research examining dose-specific and formulation-specific effects is warranted. The potential synergy of GLP-1 RAs with SGLT2 inhibitors should be systematically investigated in combination therapy trials, given complementary mechanisms such as natriuresis, hemodynamic modulation, and anti-inflammatory effects. Furthermore, exploring mechanistic differences among GLP-1 RAs, such as receptor binding affinity, anti-inflammatory signaling, and renal hemodynamic pathways, may elucidate the observed variability in cardiorenal protection. Future incorporation of RWD will be critical to validate these findings in more heterogeneous and diverse patient populations. The SUCRA-based ranking should be interpreted with caution, as it is influenced by the quantity and precision of available trial data. Agents supported by fewer studies, such as efpeglenatide, may yield less stable SUCRA estimates that reflect probabilistic ranking rather than definitive superiority. Moreover, the term *renal composite events* in this analysis refers specifically to beneficial renal outcomes (e.g., reduced progression of albuminuria, slower eGFR decline, or lower incidence of renal replacement therapy), not to adverse events.

Differences in trial design, baseline populations, and definitions of renal composites across studies may limit direct comparability and should be considered when interpreting results. Future head-to-head RCTs incorporating both CV and renal endpoints, with standardized definitions and harmonized outcome reporting, are warranted to confirm these findings. Additionally, greater use of RWD will be essential to validate these associations across more diverse patient subgroups-including those with CKD, older adults, and individuals with multimorbidity-thereby enhancing the clinical generalizability of these observations.

This study has several limitations that should be acknowledged when interpreting the findings. First, while NMA allows for indirect comparisons between agents not directly studied head-to-head, it is inherently dependent on the assumptions of transitivity and consistency, which may not fully hold across heterogeneous trial designs and populations. Variations in baseline CV or renal risk, follow-up durations, and background therapies (e.g., use of SGLT2 inhibitors) across the included trials may introduce residual confounding. Second, renal outcomes were often secondary endpoints in the original trials and varied in definition, limiting the comparability and robustness of renal-specific results. Some agents, such as cotadutide and lixisenatide, were represented by fewer studies or smaller sample sizes, resulting in wide CIs and lower SUCRA precision, which may understate or overstate their true effect. Third, the analysis did not account for dose-dependent effects or differentiate between formulations (e.g., once-weekly vs. daily dosing), which could influence efficacy and tolerability. Additionally, publication bias cannot be entirely ruled out, despite no significant findings from funnel plot asymmetry tests. A key limitation of this NMA is the inclusion of a few early-phase or small exploratory studies with incomplete reporting, which may introduce heterogeneity in quality appraisal. These studies were qualitatively summarized but not quantitatively pooled. Furthermore, no formal sensitivity or subgroup analysis was undertaken to evaluate the influence of higher-risk trials due to insufficient stratified data; however, the large-scale CVOTs dominated weighting, minimizing the influence of smaller studies. Finally, the absence of individual patient-level data restricted the ability to perform subgroup analyses based on age, renal function, or CVD history. Another limitation is that the analysis did not adjust for concomitant cardioprotective therapies such as SGLT2 inhibitors or insulin. Although these treatments were variably used across trials, consistent directional benefits of GLP-1 RAs were observed in trials both with and without background SGLT2 inhibitor therapy, suggesting that their cardiorenal effects are likely additive rather than confounded. Nonetheless, the absence of patient-level adjustment precludes definitive assessment of interaction effects.

Future research should focus on addressing the existing gaps in the comparative evaluation of GLP-1 RAs, particularly in head-to-head trials assessing both CV and renal outcomes as primary endpoints. While current evidence supports a class effect for GLP-1 RAs in reducing cardiorenal risk, individual variability in efficacy and safety profiles warrants further investigation. Future RCTs should be designed to directly compare top-performing agents, such as efpeglenatide, semaglutide, and liraglutide, across diverse populations, including those with advanced CKD, HF, or multiple comorbidities. Additionally, longer follow-up durations and standardized definitions for renal composite outcomes are essential to improve consistency and interpretability. Real-world evidence and patient-level data analyses will be valuable in evaluating the effectiveness, adherence, and tolerability of different formulations and dosages in clinical practice. Moreover, mechanistic studies exploring the pathways underlying renal and CV protection can guide the development of next-generation GLP-1 RAs or combination therapies. Future research should also investigate the potential synergistic effects of combining GLP-1 RAs with other cardioprotective agents, such as SGLT2 inhibitors, to optimize outcomes. Incorporating cost-effectiveness analyses and patient-reported outcomes can further inform guideline development and personalized treatment strategies. Ultimately, a more nuanced understanding of agent-specific benefits will enhance therapeutic decision-making in T2DM care.

## Conclusions

This comprehensive network meta-analysis provides comparative evidence on the CV and renal efficacy of injectable GLP-1 receptor agonists in patients with T2DM. The findings confirm a consistent class-wide benefit of GLP-1 RAs in reducing MACE and improving renal composite outcomes, referring to beneficial renal effects such as slower eGFR decline and reduced progression to macroalbuminuria, while also revealing inter-agent variability. Among all agents evaluated, efpeglenatide demonstrated superior efficacy, ranking highest in both CV and renal domains and showing the greatest overall reduction in MACE and renal composite outcomes based on pooled odds ratios and SUCRA rankings. However, this apparent superiority should be interpreted cautiously, as the number of contributing trials for efpeglenatide was limited, and SUCRA scores are influenced by data availability and study precision. Other GLP-1 RAs, including albiglutide, semaglutide, dulaglutide, and liraglutide, also demonstrated significant benefits, reinforcing the overall cardiorenal protective class effect of this drug category. Variations in trial design, baseline populations, and endpoint definitions may influence comparative interpretation. Future head-to-head RCTs incorporating standardized CV and renal endpoints are essential to confirm these comparative rankings. Furthermore, validation through RWD across diverse patient populations, including those with CKD, the elderly, and multimorbid individuals, will be crucial for establishing the generalizability of these findings and optimizing individualized therapeutic strategies in T2DM management.

## Appendices

Appendix A

**Table 3:** Inconsistencies in MACE outcomes:

	Comparison	No.Studies	NMA	Direct	Indirect	Difference	Diff_95CI_lower	Diff_95CI_upper	pValue
1	Albiglutide:Cotadutide	0	-0.581895105061467	NA	-0.581895105061467	NA	NA	NA	NA
2	Albiglutide:Dulaglutide	0	-0.124909357154067	NA	-0.124909357154067	NA	NA	NA	NA
3	Albiglutide:Efpeglenatide	0	0.0471894308111663	NA	0.0471894308111663	NA	NA	NA	NA
4	Albiglutide:Exenatide	0	-0.146115108124142	NA	-0.146115108124142	NA	NA	NA	NA
5	Albiglutide:Liraglutide	0	-0.104952619668088	NA	-0.104952619668088	NA	NA	NA	NA
6	Albiglutide:Lixisenatide	0	0.00527587807565399	NA	0.00527587807565399	NA	NA	NA	NA
7	Albiglutide:Placebo	1	-0.256544842232915	-0.256544842232937	NA	NA	NA	NA	NA
8	Albiglutide:Semaglutide	0	-0.0748502041367948	NA	-0.0748502041367948	NA	NA	NA	NA
9	Cotadutide:Dulaglutide	0	0.4569857479074	NA	0.4569857479074	NA	NA	NA	NA
10	Cotadutide:Efpeglenatide	0	0.629084535872633	NA	0.629084535872633	NA	NA	NA	NA
11	Cotadutide:Exenatide	0	0.435779996937325	NA	0.435779996937325	NA	NA	NA	NA
12	Cotadutide:Liraglutide	0	0.476942485393379	NA	0.476942485393379	NA	NA	NA	NA
13	Cotadutide:Lixisenatide	0	0.587170983137121	NA	0.587170983137121	NA	NA	NA	NA
14	Cotadutide:Placebo	0	0.325350262828552	NA	0.325350262828552	NA	NA	NA	NA
15	Cotadutide:Semaglutide	1	0.507044900924672	0.507044900926085	NA	NA	NA	NA	NA
16	Dulaglutide:Efpeglenatide	0	0.172098787965233	NA	0.172098787965233	NA	NA	NA	NA
17	Dulaglutide:Exenatide	0	-0.0212057509700748	NA	-0.0212057509700748	NA	NA	NA	NA
18	Dulaglutide:Liraglutide	0	0.0199567374859791	NA	0.0199567374859791	NA	NA	NA	NA
19	Dulaglutide:Lixisenatide	0	0.130185235229721	NA	0.130185235229721	NA	NA	NA	NA
20	Dulaglutide:Placebo	2	-0.131635485078848	-0.131635485078847	NA	NA	NA	NA	NA
21	Dulaglutide:Semaglutide	0	0.0500591530172723	NA	0.0500591530172723	NA	NA	NA	NA
22	Efpeglenatide:Exenatide	0	-0.193304538935308	NA	-0.193304538935308	NA	NA	NA	NA
23	Efpeglenatide:Liraglutide	0	-0.152142050479254	NA	-0.152142050479254	NA	NA	NA	NA
24	Efpeglenatide:Lixisenatide	0	-0.0419135527355122	NA	-0.0419135527355122	NA	NA	NA	NA
25	Efpeglenatide:Placebo	2	-0.303734273044081	-0.303734273044074	NA	NA	NA	NA	NA
26	Efpeglenatide:Semaglutide	0	-0.122039634947961	NA	-0.122039634947961	NA	NA	NA	NA
27	Exenatide:Liraglutide	0	0.0411624884560539	NA	0.0411624884560539	NA	NA	NA	NA
28	Exenatide:Lixisenatide	0	0.151390986199796	NA	0.151390986199796	NA	NA	NA	NA
29	Exenatide:Placebo	1	-0.110429734108773	-0.110429734108776	NA	NA	NA	NA	NA
30	Exenatide:Semaglutide	0	0.0712649039873471	NA	0.0712649039873471	NA	NA	NA	NA
31	Liraglutide:Lixisenatide	0	0.110228497743742	NA	0.110228497743742	NA	NA	NA	NA
32	Liraglutide:Placebo	3	-0.151592222564827	-0.151592222564822	NA	NA	NA	NA	NA
33	Liraglutide:Semaglutide	0	0.0301024155312932	NA	0.0301024155312932	NA	NA	NA	NA
34	Lixisenatide:Placebo	1	-0.261820720308569	-0.261820720308579	NA	NA	NA	NA	NA
35	Lixisenatide:Semaglutide	0	-0.0801260822124489	NA	-0.0801260822124489	NA	NA	NA	NA
36	Semaglutide:Placebo	4	-0.18169463809612	-0.181694638096117	NA	NA	NA	NA	NA

Appendix B

**Table 4:** Inconsistencies in Renal adverse event outcomes

	Comparison	No.Studies	NMA	Direct	Indirect	Difference	Diff_95CI_lower	Diff_95CI_upper	pValue
1	Albiglutide:Cotadutide	0	-0.206965935924233	NA	-0.206965935924233	NA	NA	NA	NA
2	Albiglutide:Dulaglutide	0	0.0210840523840771	NA	0.0210840523840771	NA	NA	NA	NA
3	Albiglutide:Efpeglenatide	0	0.269111228015206	NA	0.269111228015206	NA	NA	NA	NA
4	Albiglutide:Exenatide	0	-0.00279011068968382	NA	-0.00279011068968382	NA	NA	NA	NA
5	Albiglutide:Liraglutide	0	0.101200856443849	NA	0.101200856443849	NA	NA	NA	NA
6	Albiglutide:Lixisenatide	0	-0.143113200898269	NA	-0.143113200898269	NA	NA	NA	NA
7	Albiglutide:Placebo	1	-0.142778024089366	-0.142778024089367	NA	NA	NA	NA	NA
8	Albiglutide:Semaglutide	0	-0.0511160203440152	NA	-0.0511160203440152	NA	NA	NA	NA
9	Cotadutide:Dulaglutide	0	0.22804998830831	NA	0.22804998830831	NA	NA	NA	NA
10	Cotadutide:Efpeglenatide	0	0.476077163939439	NA	0.476077163939439	NA	NA	NA	NA
11	Cotadutide:Exenatide	0	0.20417582523455	NA	0.20417582523455	NA	NA	NA	NA
12	Cotadutide:Liraglutide	0	0.308166792368083	NA	0.308166792368083	NA	NA	NA	NA
13	Cotadutide:Lixisenatide	0	0.0638527350259646	NA	0.0638527350259646	NA	NA	NA	NA
14	Cotadutide:Placebo	0	0.0641879118348674	NA	0.0641879118348674	NA	NA	NA	NA
15	Cotadutide:Semaglutide	1	0.155849915580218	0.155849915580218	NA	NA	NA	NA	NA
16	Dulaglutide:Efpeglenatide	0	0.248027175631129	NA	0.248027175631129	NA	NA	NA	NA
17	Dulaglutide:Exenatide	0	-0.0238741630737609	NA	-0.0238741630737609	NA	NA	NA	NA
18	Dulaglutide:Liraglutide	0	0.0801168040597722	NA	0.0801168040597722	NA	NA	NA	NA
19	Dulaglutide:Lixisenatide	0	-0.164197253282346	NA	-0.164197253282346	NA	NA	NA	NA
20	Dulaglutide:Placebo	1	-0.163862076473443	-0.163862076473443	NA	NA	NA	NA	NA
21	Dulaglutide:Semaglutide	0	-0.0722000727280924	NA	-0.0722000727280924	NA	NA	NA	NA
22	Efpeglenatide:Exenatide	0	-0.271901338704889	NA	-0.271901338704889	NA	NA	NA	NA
23	Efpeglenatide:Liraglutide	0	-0.167910371571356	NA	-0.167910371571356	NA	NA	NA	NA
24	Efpeglenatide:Lixisenatide	0	-0.412224428913474	NA	-0.412224428913474	NA	NA	NA	NA
25	Efpeglenatide:Placebo	1	-0.411889252104572	-0.411889252104571	NA	NA	NA	NA	NA
26	Efpeglenatide:Semaglutide	0	-0.320227248359221	NA	-0.320227248359221	NA	NA	NA	NA
27	Exenatide:Liraglutide	0	0.103990967133533	NA	0.103990967133533	NA	NA	NA	NA
28	Exenatide:Lixisenatide	0	-0.140323090208585	NA	-0.140323090208585	NA	NA	NA	NA
29	Exenatide:Placebo	1	-0.139987913399682	-0.139987913399682	NA	NA	NA	NA	NA
30	Exenatide:Semaglutide	0	-0.0483259096543314	NA	-0.0483259096543314	NA	NA	NA	NA
31	Liraglutide:Lixisenatide	0	-0.244314057342118	NA	-0.244314057342118	NA	NA	NA	NA
32	Liraglutide:Placebo	1	-0.243978880533215	-0.243978880533215	NA	NA	NA	NA	NA
33	Liraglutide:Semaglutide	0	-0.152316876787865	NA	-0.152316876787865	NA	NA	NA	NA
34	Lixisenatide:Placebo	1	0.000335176808902771	0.00033517680890298	NA	NA	NA	NA	NA
35	Lixisenatide:Semaglutide	0	0.0919971805542535	NA	0.0919971805542535	NA	NA	NA	NA
36	Semaglutide:Placebo	2	-0.0916620037453507	-0.0916620037453506	NA	NA	NA	NA	NA
